# Tailored Synthesis and Profiling of Capped Silver and Selenium Nanoparticles for Topical Applications in Paediatric Dentistry

**DOI:** 10.3390/dj13100456

**Published:** 2025-10-06

**Authors:** Amjad Almuqrin, Chaminda Jayampath Seneviratne, Laurence J. Walsh, Sobia Zafar

**Affiliations:** 1 School of Dentistry, The University of Queensland, Brisbane, QLD 4006, Australia; jaya.seneviratne@uq.edu.au (C.J.S.); l.walsh@uq.edu.au (L.J.W.); 2 School of Dentistry, The University of Hail, Hail 2440, Saudi Arabia

**Keywords:** nanotechnology, metal nanoparticles, silver nanoparticles, selenium nanoparticles, nanoparticles synthesis, biocompatible capping agents, paediatric dentistry

## Abstract

**Background:** Silver fluoride medicaments effectively arrest caries progression but cause permanent staining. Nanoparticles are increasingly utilised in paediatric dentistry due to their antimicrobial properties. **Aim:** This study reports the synthesis and characterisation of silver and selenium nanoparticles stabilised with natural agents. **Methods:** Six silver and selenium nanoparticles were chemically synthesised and stabilised using biocompatible eco-friendly capping agents, including casein, bovine serum albumin, chitosan, citrate, and tannic acid. Characterisation was performed using Tyndall scattering, UV-Vis spectroscopy, transmission electron microscopy, and ICP-OES. **Results:** The synthesised particles were spherical in shape, ranging in size from 2.5 to 24 nm for silver and 35 to 43 nm for selenium. Elemental analysis confirmed the absence of heavy metals. **Conclusions**: These findings highlight the successful synthesis of capped silver and selenium nanoparticles. The observed characteristics suggest potential relevance for future antimicrobial applications in paediatric dentistry.

## 1. Introduction

Early childhood caries (ECC) is a leading childhood illness as well as a significant cause of avoidable hospital admissions [1]. Managing ECC can be complex due to the young age of affected patients and the rapid, aggressive nature of the disease. Conventional treatments typically involve operative interventions such as tooth preparation, restorations, and pulp therapy, or surgical procedures such as extractions. These interventions often necessitate a high level of child cooperation and may require general anaesthesia when performed on uncooperative patients.

Silver fluoride (SF) topical solutions effectively arrest caries progression in young children who may not yet comply with standard treatments, which minimises the need for invasive procedures [2]. However, the ionic silver component in these solutions causes permanent tooth staining, raising aesthetic concerns among parents and dental practitioners, limiting their acceptance as a first line treatment option [3].

In recent years, metal-based nanomaterials have attracted attention in dentistry due to their potent antimicrobial properties, tuneable physicochemical characteristics, and ability to disrupt oral biofilms [4]. Nanoparticles (NPs), with their ultrafine size (1–100 nm) and high surface-to-volume ratio, exhibit superior reactivity, facilitating strong interactions with oral microbes [5]. This enables the effective eradication of oral pathogens, and penetration into biofilms [6,7]. Among the various nanomaterials explored, silver NPs (AgNPs) are particularly notable for their broad-spectrum antibiofilm efficacy, supporting their integration into dental composites [8], adhesives [9], and implants [10]. Selenium NPs (SeNPs), although less extensively studied in dentistry, are emerging as promising biocompatible antimicrobial agents [11], with additional functions in antioxidant defence and immune modulation [12,13]. The growing interest in AgNPs and SeNPs highlights the broader potential of nanotechnology to enhance antimicrobial efficacy in the oral environment while maintaining compatibility with host tissues.

The stability of NPs remains a significant challenge as they tend to agglomerate or aggregate post-synthesis, which reduces bioavailability and antimicrobial efficacy. Agglomeration, driven by weak van der Waals forces, forms loosely bound clusters that retain surface area but hinder biofilm penetration and ion release [14]. In contrast, aggregation involves strong chemical bonding, forming fused structures with significantly reduced surface area and reactivity [15]. Both processes impair NP performance, necessitating effective stabilisation strategies to preserve their antimicrobial and bioactive potential in clinical applications.

Capping plays a key role by stabilising NPs and enhancing their biological actions. For instance, casein, bovine serum albumin (BSA), chitosan, citrate, and tannic acid have been shown to be effective capping agents due to their biocompatibility, cost-effectiveness, functional benefits, and scalability for clinical applications. All of the foregoing capping agents are natural compounds with low toxicity. Casein and BSA provide a protein-based stabilisation matrix, which improves NP dispersion and cellular uptake [16]. Chitosan exerts antimicrobial actions, and the charge modifications it causes enhance cellular interactions [17]. Citrate stabilises NPs, to maintain colloidal stability [18]. Tannic acid, a polyphenol with antioxidant properties, further enhances NP stability and biological performance [19]. Therefore, the present study incorporated casein, BSA, chitosan, citrate, and tannic acid as capping agents.

**Figure 1:**
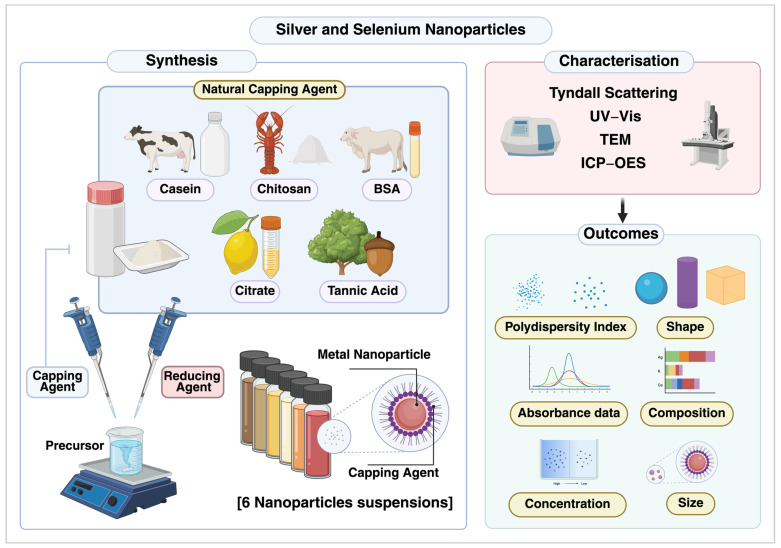
Schematic representation of the synthesis, characterisation, and outcomes of silver and selenium nanoparticles capped with naturally derived agents.

The aim of the study was to synthesise and characterise capped silver and selenium NPs for potential topical antimicrobial applications in paediatric dentistry. Specifically, we assessed their physiochemical characteristics and elemental composition. The graphical illustration is shown in Figure 1.

## 2. Materials and Methods

### 2.1. Preparation of NPs

**Figure 2:**
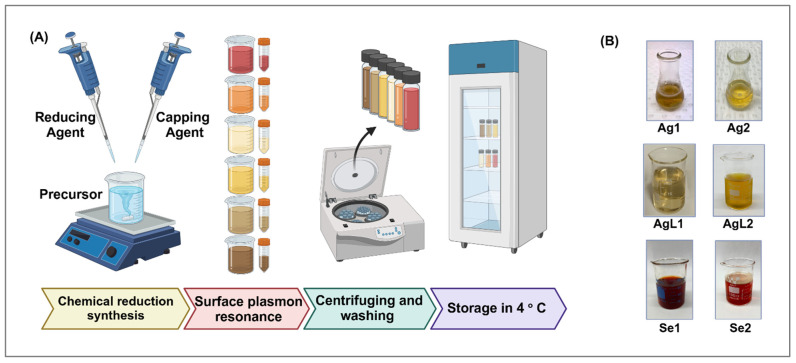
Illustration of the major chemical synthesis stages of nanoparticles. (**A**) Diagram explaining the key stages of chemical synthesis. (**B**) Post-synthesis images of nanoparticles (NPs), showing a colour change due to the SPR phenomenon.

Based on published methods in the literature, six silver and selenium NPs were synthesised chemically, i.e., four AgNPs and two SeNPs (Table 1). The synthesis process involved a chemical reduction approach, where a precursor solution was treated with a reducing agent and a capping agent to facilitate nanoparticle formation, followed by centrifugation, washing, and storage at 4 °C. An overview of the synthesis stages and the final appearance of the NPs is illustrated in Figure 2. Notably, the distinct colour changes are attributable to the surface plasmon resonance (SPR) phenomenon. The SPR is highly sensitive to particle size, shape, and dielectric environment, confirming successful nanoparticle formation and morphological changes.

Ag1: The Ag1 synthesis method was adapted from previous work [20], employing a chemical reduction approach. In brief, 3.0 mL of 0.6% *w*/*v* casein (Sigma-Aldrich, St. Louis, MO, USA) and 0.1 mL of 1.0 M NaOH (Sigma-Aldrich, St. Louis, MO, USA) were combined in a 25 mL glass flask. After 10 min of gentle magnetic stirring at room temperature, 14.9 mL of deionised (DI) water was added, after which the mixture was heated to 60 °C and stirred for 5 min. Lastly, 2 mL of 20 mM silver nitrate (AgNO_3_) (Sigma-Aldrich, St. Louis, MO, USA) was gradually added dropwise to the mixture and the solution magnetically stirred for 60 min at 60 °C. During this time, the solution colour changed from transparent to golden yellow. Subsequently, the solution was centrifuged at 5000 rpm at 4 °C and washed three times with DI water, with the pellet retained and the supernatant discarded at each washing step. The Ag1 NPs were then resuspended in DI water, transferred to light-proof sterile vials, and stored at 4 °C. The same approach was used for Ag2 synthesis, substituting casein with BSA (0.6% *w*/*v*) (Sigma-Aldrich, St. Louis, MO, USA). The final solution was, again, a vivid yellow colour.

AgL1: The synthesis method was modified from past work [21], using 70 °C rather than 100 °C. In brief, 500 mL of DI water was heated to 70 °C in a 2 L beaker on a magnetic stirrer. To this was added 0.1 g of AgNO_3_ (Sigma-Aldrich, St. Louis, MO, USA). After mixing for 5 min, 0.1 g of trisodium citrate dihydrate (Sigma-Aldrich, St. Louis, MO, USA) dissolved in 10 mL of DI water and added dropwise with magnetic stirring. The solution was heated to 94 °C and mixing was undertaken in the dark for 2.5 h, with the beaker covered to minimise evaporative water loss. By the end of this period, the solution was a faint yellow colour. Stirring continued for a further 1 h while the solution cooled to room temperature. The final solution was decanted into a brown glass bottle and stored at room temperature.

AgL2: The sample was synthesised following a modification of the method of Bastus et al. (2014) [22]. In brief, 100 mL of DI water was heated to 80 °C and divided into equal portions of 50 mL. To the first, 0.147 g trisodium citrate (Sigma-Aldrich, St. Louis, MO, USA) was added to give a concentration of 5 mM, followed by 4.25 mg of tannic acid (Sigma-Aldrich, St. Louis, MO, USA), giving a concentration of 0.025 mM. A second solution of 0.0425 g of AgNO_3_ (Sigma-Aldrich, St. Louis, MO, USA) in 10 mL of DI water was prepared (25 mM). This was then preheated in a microwave to 80 °C and added in one step to the first solution. The mixture was kept at 80 °C under vigorous stirring. The solution turned bright yellow immediately, indicating nanoparticle formation. Stirring continued for a further 1 h while solution cooled to room temperature. The final solution was decanted into a brown glass bottle and stored at room temperature.

Se1 and Se2: These NPs were synthesised using a previously described method [23] with minor modifications. Initially, a stock solution of 1% *wt*/*v* chitosan (Sigma-Aldrich, St. Louis, MO, USA) was prepared in 1% (*v*/*v*) acetic acid (Millipore Corporation, Summit Drive, MA, USA). Then, 2 mL of the chitosan solution was mixed with 8 mL of 10 mM Na_2_SeO_3_ (Sigma-Aldrich, St. Louis, MO, USA), and the mixture was magnetically stirred for 10 min at room temperature. Subsequently, 4 mL of ice-cold 0.1 M sodium borohydride (NaBH_4_) (Sigma-Aldrich, St. Louis, MO, USA) was added gradually and the mixture was stirred continuously for 60 min. The solution became dark red in colour. The NPs were washed three times with DI water by centrifugation at 14,000 rpm. Finally, the NPs were stored in light-proof sterile tubes at 4 °C.

A similar protocol was used for Se2, using BSA in water instead of chitosan in acetic acid. A 2 mL volume of 1% *w*/*v* BSA solution was mixed with 8 mL of 10 mM Na_2_SeO_3_ solution (Sigma-Aldrich, St. Louis, MO, USA). Then, 4 mL of ice-cold 0.1 M NaBH_4_ was added dropwise. An orange colour developed as the SeNPs were formed. As before, the SeNPs were centrifuged, washed twice with ultra-pure water and stored at 4 °C.

### 2.2. Characterisation

**Figure 3:**
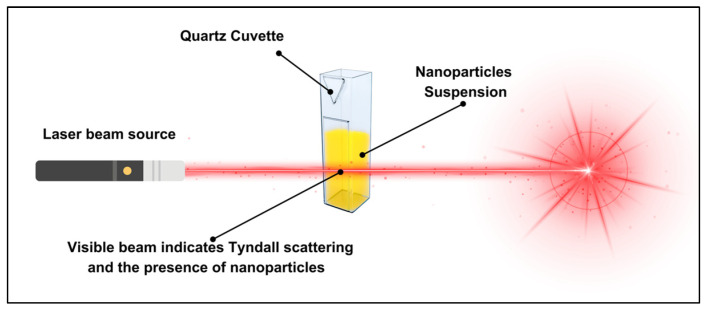
Schematic representation of the Tyndall effect in a NPs suspension. A laser beam passes through a quartz cuvette containing NPs, producing visible scattering that confirms their presence.

Tyndall scattering: The Tyndall effect is a light-scattering phenomenon utilised to promptly confirm NP formation in colloidal solutions. When an intense light source, such as a laser beam, passes through the suspension, the NPs scatter the light, producing a visible path (Figure 3). The extent of scattering varies with particle size and the wavelength of the incident light, with smaller NPs scattering more effectively at shorter wavelengths.

In this study, three handheld laser pointers of different wavelengths were used: violet (405 ± 10 nm), green (532 ± 10 nm), and red (670 ± 10 nm), each with a maximum power output of <1000 mW (Memory Tech, Sydney, NSW, Australia). All three laser systems were rated as class IIIa, with parallel beams (less than 2 milliradian divergence), operating in continuous mode with a spot size of 2 mm and a power of 1 mW for 405 nm, 4 mW for 532 nm, and 1 mW for 650 nm. Laser power was measured using a laser power metre (SpectraMet, Laserdyne Technologies, Molendinar, QLD, Australia). A 1 mL aliquot of each NP suspension was dispersed in a 3.5 mL glass cuvette, and the laser beams were directed horizontally through the dispersion at a consistent distance of 5 cm from the cuvette’s surface to maintain a uniform light path. Deionised (DI) water was used as a negative control to compare scattering effects in the absence of NPs. Afterward, three images of each NP suspension were captured using a smartphone camera (model: Iphone 15 Pro; Apple Inc., Cupertino, CA, USA) positioned perpendicular to the laser path. The experiment was performed in a light-controlled dark setting to minimise external light reflections. The observed scattering patterns were qualitatively analysed, to determine the presence of NPs following the synthesis process. This test was conducted under safety protocols for class IIIa lasers which included wearing laser safety goggles and a portable laser stand to restrict the beam path and minimise exposure risks.

UV-Visible spectroscopy: The ultraviolet–visible (UV-Vis) absorbance spectra of NP suspensions were measured using a spectrophotometer (GENESYS™ 10S UV-Vis spectrophotometer, Thermo Fisher Scientific, Waltham, MA, USA) immediately after NP synthesis. Prior to measurement, samples were vortex mixed and ultrasonicated for 5 min to ensure NP dispersion, before being transferred into 3.5 mL quartz cuvettes for analysis. Absorbance spectra were collected within the UV–visible range, with deionised (DI) water used as the baseline. Aqueous solutions of the capping agents were also analysed separately under the same conditions for reference.

Transmission Electron Microscopy (TEM): Six NP samples were transferred to the Central Analytical Research Facility (CARF) of the Queensland University of Technology (QUT). The samples were vortex mixed and ultrasonicated for at least 5 min to ensure a homogeneous dispersion. Subsequently, the samples were drop mounted onto 300 mesh lacey formvar/carbon-coated copper grids. The imaging process utilised a JEOL JEM-2100 TEM (JEOL Ltd., Tokyo, Japan), operated at 200 kV, with a 4K Fx416 TVIPS CCD camera. The TEM micrographs were then processed through ImageJ software (version 1.47b, NIH, Bethesda, MD, USA) for analysis. The diameter measurements of each sample involved the analysis of at least 100 separate particles which were chosen from different non-overlapping fields to achieve representative data.

Inductively Coupled Plasma Optical Emission Spectroscopy (ICP-OES): Elemental concentrations of NPs were determined using a PerkinElmer 8300DV ICP-OES (PerkinElmer, Waltham, MA, USA) fitted with an ESI SC-4DX autosampler. ICP-OES was used to determine the overall concentration of silver and selenium NPs, as conventional drying and weighing methods were impractical due to the presence of organic capping agents. Drying the samples could alter their chemical properties, as the organic capping agents might degrade or undergo structural changes, affecting NP stability. This analysis allowed experiments to be designed based on Ag or Se concentrations, independent of capping agents. ICP-OES was also used to screen for trace contaminants of heavy metals, such as lead and cadmium.

Six aqueous nanomaterial samples were diluted in 2% ultra-pure nitric acid at a 1:10 dilution factor. Multi-element quantitation was performed using the PerkinElmer 8300DV ICP-OES, equipped with an ESI SC-4DX autosampler (Elemental Scientific Inc., Omaha, NE, USA) and a PrepFAST 2 sample handling unit (Elemental Scientific Inc., Omaha, NE, USA) for online internal standardisation and auto-dilution of samples and calibration standards. Sub-boiling distilled nitric acid was used to prepare all standards and blank solutions. Instrument calibration was conducted using external standardisation, with multi-element standards prepared in-house from ICP-MS grade single-element stock solutions (High Purity Standards, Charleston, SC, USA). A combination of axial and radial viewing modes was used, with radial mode specifically employed for alkali metals (Li, Na, K). Method robustness, accuracy, and precision were verified through the continuous analysis of multiple Certified Reference Materials (CRMs), covering a range of common matrices and analyte concentrations (National Institute of Standards and Technology, Gaithersburg, MD, USA; United States Geological Survey, Reston, VA, USA).

## 3. Results

**Figure 4:**
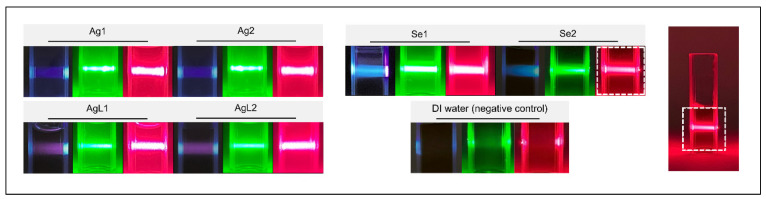
Tyndall scattering in NP suspensions using different laser wavelengths (405, 532, and 650 nm). Scattering was observed in all samples except the DI water control, with intensity variations suggesting differences in particle properties.

Tyndall scattering: All NP suspensions exhibited visible light Tyndall scattering when exposed to laser beams at 405 nm, 532 nm, and 670 nm, confirming the presence of suspended NPs, while the DI water negative control showed no scattering, as shown in Figure 4. As this test was merely qualitative, no interpretation of the scattering intensity between wavelengths or samples was attempted.

**Figure 5:**
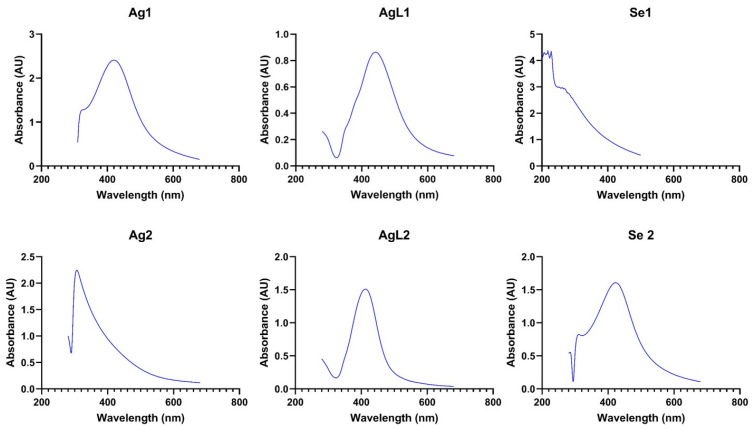
Absorbance curves of the synthesised NPs.

UV-Visible spectroscopy: As illustrated in Figure 5, absorbance spectra confirmed the formation of silver and selenium NPs. Ag1, AgL1, and AgL2 exhibited distinct extinction bands with λmax at 420 nm, while Se2 showed an extinction band with λmax at 410 nm. The absorbance spectrum of Ag2 showed a broader extinction band, with no well-defined λmax, and Se1 exhibited a similarly broad absorbance curve without a sharp peak.

**Figure A1:**
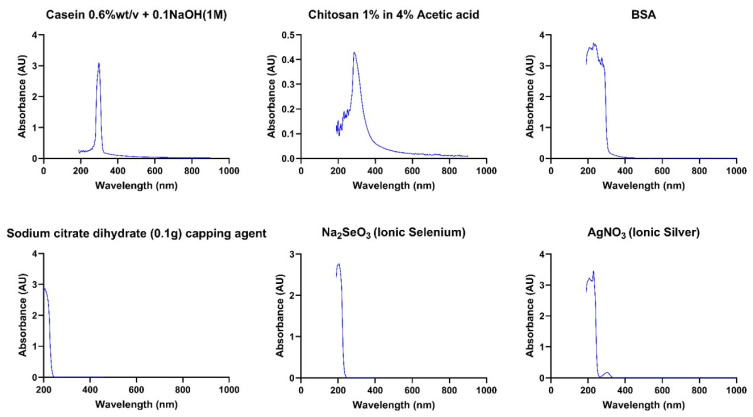
Absorbance curves of the capping agents and precursors.

The well-defined λmax at 420 nm in Ag1, AgL1, and AgL2 corresponds to the known SPR of silver NPs, while the broader extinction bands in Ag2 and Se1 suggest the influence of BSA and chitosan capping agents, potentially broadening the absorbance curves and affecting NP dispersion and size distribution. The λmax at around 400 nm in Se2 indicates a more uniform NP size, whereas the broader extinction band in Se1 suggests greater size variation. Absorbance curves of the capping agents and precursors are illustrated in Figure A1 in Appendix A.

**Figure 6:**
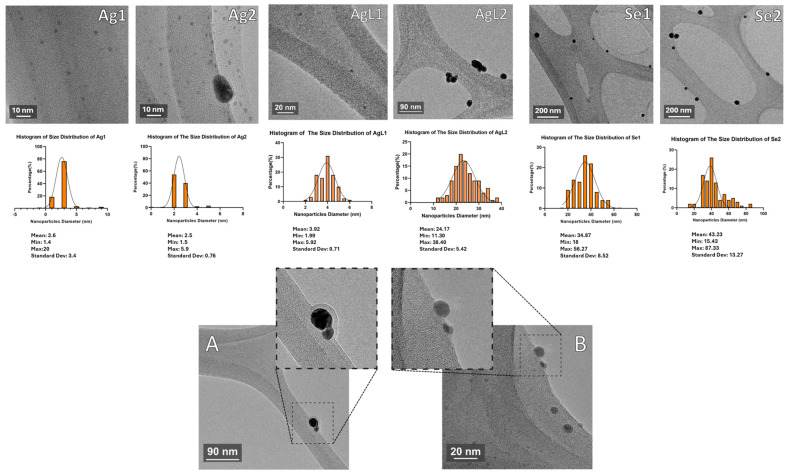
TEM images of the synthesised NPs, along with histograms illustrating their size distribution. (**A**) NPs are capped, which appear as a metal core structure with a definite external layer. (**B**) Uncapped NPs, which were prepared in separate work, display bare metal cores with no outer membrane and are shown here for comparison.

TEM: TEM images are shown in Figure 6. These revealed that the synthesised NPs were predominantly spherical in shape, with the following average diameters (means ± standard deviations): Ag1—3.6 nm + 3.4; Ag2—2.5 nm + 0.8, AgL1—3.92 nm + 0.71, AgL2—24.17 nm + 5.42; Se1—34.9 nm + 8.5; and Se2—43.2 nm + 13.2.

**Figure 7:**
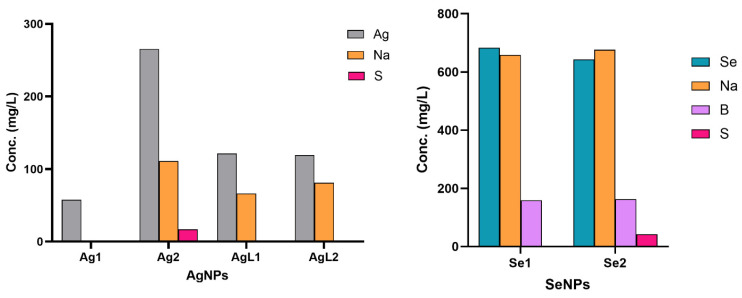
Elemental composition of AgNPs and SeNPs determined by ICP-OES.

ICP-OES: Elemental analysis confirmed successful NP synthesis (Table 2 and Figure 7). Ag concentrations were 57.44 mg/L for Ag1, 265.8 mg/L for Ag2, 121.6 mg/L for AgL1, and 119.2 mg/L for AgL2. Similarly, Se concentrations were 683.1 mg/L for Se1 and 642.8 mg/L for Se2. These measured values align with expected synthesis conditions. No detectable contamination with heavy metals such as lead (Pb) or cadmium (Cd) was observed, confirming the purity of the synthesised NPs. Trace amounts of chromium were detected in the Se1 sample.

## 4. Discussion

Aiming at exploring plausible alternatives or augmentative options for ionic silver in SF formulations, six silver and selenium NPs were prepared utilising naturally sourced stabilising agents. The ultimate intention of this research pathway is to develop topical products, particularly aqueous solutions, that could be applied to arrest dental caries lesions in children [11]. Considering our previous work, we hypothesise that NPs may have the potential to minimise, or even prevent, colour change [11]. However, post application colour change will need to be assessed in a dedicated separate study.

The fabrication process was verified using rapid, onsite methods (Tyndall scattering and UV-Vis’s spectroscopy) and further characterised in terms of particle size, morphology, and elemental composition via TEM and ICP-OES, respectively. AgNPs ranged from 2.5 to 24 nm, while SeNPs ranged from 35 to 43 nm. UV-Vis and TEM analyses validated successful NP preparation and spherical morphology. ICP-OES quantified the elemental composition and concentration. Furthermore, it confirmed the absence of toxic heavy metals. Of particular interest, protein-capped AgNPs, particularly the BSA-stabilised Ag2, exhibited the smallest and most uniform size distribution. In combination, these findings suggest that capped metal NPs could provide improved long-term stability and represent a practical topical antimicrobial choice for caries arrest in paediatric dentistry.

The selection of AgNPs and SeNPs as potential antimicrobial and cariostatic agents was guided by their high antimicrobial potency [24], biocompatibility when used at appropriate concentrations [25], extended shelf life if properly stabilised [26], and low sensitivity to light [27]. While ionic silver is well established in antimicrobial applications, the transition to nanoparticulate forms offers enhanced bioactivity; however, it introduces new challenges; specifically, agglomeration and instability under physiological conditions. Such instability can compromise their effectiveness and safety, ultimately shorten their shelf life and limit their clinical utility [28]. To address these issues, the application of suitable stabilisers to metal NPs has become a widely adopted strategy. By preventing uncontrolled NP growth, it can enhance NP reactivity and stability and maintain bioactivity, thereby supporting antimicrobial performance comparable to that of SF formulations.

Building upon this rationale and recognising that SF formulations currently used in dentistry are water-based, a similar aqueous medium was adopted for AgNPs synthesis. Additionally, using an aqueous formulation should improve biocompatibility by reducing the need for organic solvents. Residues of such solvents may introduce toxicity concerns in clinical settings. Chemical synthesis provides a straightforward and scalable approach for NP fabrication and allows for the incorporation of capping agents [29]. As a controlled, reproducible approach, it provides consistency and batch-to-batch uniformity, provided there is precise control of the reaction conditions, including the temperature and time, over prolonged periods. The cost of reagents must also be considered in terms of large-scale production.

Following the successful synthesis, morphological analysis was carried out to assess the physical properties of the generated NPs. The TEM analysis revealed variations in NP sizes, which could influence their antimicrobial activity. Smaller NPs generally exhibit greater bioactivity due to their higher surface area-to-volume ratio, and increased ability to physically penetrate complex microbial membranes. Among the AgNPs, Ag1, Ag2, and AgL1 had small sizes (<5 nm), and Ag2 (BSA-capped) had the narrowest size distribution. In contrast, NPs of AgL2 (citrate and tannic acid-capped) were significantly larger (24.17 ± 5.42 nm), which may influence their interactions with bacterial biofilms. There is a well-established correlation between NP size and antimicrobial behaviour, where reduced dimensions enhance diffusion, membrane disruption, and intracellular interactions thereby boost antimicrobial actions [30].

The SeNPs were notably larger than the AgNPs and exhibited size variations according to the choice of capping agent, with Se1 (chitosan-capped) at 34.9 ± 8.5 nm and Se2 (BSA-capped) at 43.2 ± 13.2 nm. The larger size of SeNPs versus AgNPs may lower their effectiveness as antimicrobial agents, and this aspect remains to be explored. Given that size directly affects the extent of microbial contact and reactive surface exposure, optimising SeNP dimensions through refined synthesis methods could enhance their therapeutic applicability. Several strategies can optimise the fabrication protocol to achieve smaller SeNPs, which include elevating the reaction temperature, adjusting the pH of the reaction medium, altering the capping agent or utilising a stronger reducing agent.

Hydrodynamic size and zeta potential data were not used in this study, as particle size was determined from electron micrographs, which directly visualise the electron-dense silver or selenium cores. This approach was preferred because our focus was on the exact metallic nanoparticle dimensions, rather than the hydrodynamic diameter that also reflects the surrounding capping agent layer, which varied substantially in molecular size across the agents used.

In addition to particle size, nanoparticle shape is central to modulating biological interactions including biodistribution, cellular uptake, and cytotoxicity. In general, spherical NPs exhibit lower cytotoxicity compared with anisotropic forms such as rods [31], sheets [32], prisms [33], and needles [34]. Accordingly, the synthesis strategy adopted in this study prioritised the formation of spherical NPs, with the goal being to minimise toxicity while preserving antimicrobial potency. The TEM images confirmed that the NPs exhibited a predominantly spherical morphology. The biological safety of the formulations needs confirmation through cytotoxicity assays, which were not included in this study but will be a component of future research.

The spherical shape of NPs probably reflects the thermodynamic for minimal surface energy in the aqueous synthesis medium. Moderate to high temperatures, as followed here, promote NP isotropic growth where nucleation rate and surface diffusion are rapid. Thus, NPs form equally in all directions in a symmetric and uniform sphere shape [35]. Another factor controlling shape is employing the optimal capping agent which binds evenly to the NP surface, prevents anisotropy and maintains the sphericity. Morphology is highly relevant, as spherical NPs are associated with reduced toxicity to human cells such as epithelial cells of gingival tissue. Addressing NP shape is advantageous in paediatric dental application, where biocompatibility is a cornerstone requirement. Further studies are needed to explore the toxicity of the various NPs, to select those with optimal biological performance and low toxicity.

Spectroscopy data validated the formation of both silver and selenium nanoparticles through characteristic SPR absorbance patterns. Three of the four AgNPs showed a sharp peak between 400 and 450 nm, which aligns with the literature on the 420 nm peak [20,21,22,36]. These peaks are indicators of collective oscillations of conduction electrons on the silver NP surface when excited by incident light, which is a phenomenon highly sensitive to particle size, shape, dielectric environment, and interparticle spacing. The Ag2 sample, which was capped with BSA, was blue shifted and had a peak absorbance at a lower wavelength, which might indicate some absorbance by the BSA in the visible violet or ultraviolet range, or particle size variations [37]. A similar strong absorbance by the capping agent, which dominates over the light absorbance by the nanoparticles, likely explains the observed broad absorbance of the chitosan-capped samples of Se1. There is also the possibility that Se1 NPs have a broad size range due to the polymeric nature of chitosan as a stabiliser. In addition, the oxidation state of selenium could influence λmax positioning. Se2 peaked sharply at approximately 400 nm, a spectral signature characteristic of elemental selenium nanoparticles, indicating a relatively uniform particle population. Taken together, these optical characterisations not only confirmed NP formation but also highlighted the influence of capping agents on their spectral behaviour as further supported by compositional analysis.

Ghodake et al. reported casein peptide–capped AgNPs of ~10 ± 5 nm synthesised through the hydrolysis of casein [20]. Their method, which was also adapted for use in the present study, relied on the activation of casein-derived functional groups with aqueous NaOH to reduce Ag^+^ ions. In our work, however, the use of intact casein in Ag1 appeared to provide a denser stabilising matrix, likely resulting in smaller core sizes. Spectroscopic analysis showed an SPR maximum for Ag1 at 420 nm, whereas Ghodake et al. reported a narrower band centred at ~410 nm. Ag2 was prepared using a parallel approach but yielded much smaller particles; however, its absorbance spectrum was broader which suggests greater polydispersity. While casein is an effective stabilising agent, it is also an identified dietary allergen for some individuals; therefore, any future development of AgNP formulations incorporating casein should include appropriate consumer warnings. The AgL1 method is the simplest for synthesis because it uses citrate reduction under water heating without requiring protein stabilisers or expensive reagents. The method enables large-scale production of stable NPs through a straightforward process that requires few steps.

Yin et al. have reported the synthesis of PEG-capped AgNPs by one-step reduction of silver acetate with thiolated polyethylene glycol (PEG-SH) in ethanol, with PEG serving as both a reductant and steric stabiliser [38]. The dispersion was subsequently solvent exchanged into water, and colloidal stability in 2.5% NaF was maintained for 18 months. In contrast, the present work employed the aqueous chemical reduction of AgNO_3_ under alkaline conditions, using a designated biocompatible capping agent, and extended the experiments to include SeNP synthesis via NaBH_4_ reduction. Upon characterisation, Yin’s PEG-AgNPs were monodisperse, spherical, and ultra-small (2.56 ± 0.43 nm) with a markedly absent UV–Vis plasmon band. Our AgNPs were also predominantly spherical but spanned a wider, capping agent-dependent size range (≈2.5–24 nm) with well-defined SPR maxima for citrate and casein stabilised samples, and a broadened band for BSA-stabilised AgNPs. Both studies used TEM for the direct sizing of NPs, while ICP-OES in the present work additionally quantified elemental composition.

Preparing high purity batches of nanoparticles is essential, as the presence of trace contaminants may trigger agglomeration, promote uncontrolled redox reactions, and jeopardise chemical stability over time. Interestingly, none of the studies that were referenced for the synthesis processes had conducted a comprehensive elemental analysis on their samples, unlike the present investigation.

The elemental composition analysis confirmed successful NP synthesis and a lack of heavy metal contaminants (such as Pb). ICP-OES is a highly sensitive technique that will detect ions leached from vials that are used to store nanosuspensions. Minor carryover of sodium (from glass and from sodium borohydride) and boron (from borohydride) was detected. Sulphur in Se2 reflects cysteine and methionine amino acids in BSA. The higher sulphur content in Se2 compared with Ag2 is consistent with its use of 1% BSA, versus 0.6% BSA in Ag2, indicating a greater protein-derived contribution, rather than contamination. Some trace elements were detected in Ag2, such as Zn, which likely originated from the glass storage containers rather than from the synthesis reagents. Furthermore, trace Fe in Se1 is chemically expected. The primary amine and hydroxyl groups in chitosan effectively bind Fe^3+^ ions at acidic pH levels [39]. According to our Se1 workflow, chitosan was dissolved in a 1% acetic acid solution which might result in capturing trace amounts of Fe impurities from biopolymers and reagents as well as from labware corrosion under weakly acidic conditions. The Fe chelates compound remains stable during washing procedures before nitric acid digestion for ICP-OES testing, which produces a minimal Fe signal. In contrast, the BSA-capped (Se2) neutral aqueous route lacks this strong Fe-binding/acidic environment, so Fe remains <LOR, explaining its absence from the other samples.

Furthermore, the Se1 sample contained chromium residue which could stem from metal contaminants present in the chitosan precursor or background substance chelation. The levels of contaminants were close to the detection limits so the findings should be treated with caution.

## 5. Conclusions

The synthesis of AgNPs and SeNPs was achieved, incorporating various naturally derived capping agents to enhance nanoparticle stability. Key physicochemical properties were characterised. This lays the groundwork for further investigation into their antimicrobial efficacy.

## Figures and Tables

**Table 1:** Custom-synthesised metal NPs and their starting concentrations.

	NPs	Synthesis Concentration (μg/mL)
1	Silver—casein-capped (Ag1)	55.4
2	Silver—BSA-capped (Ag2)	265.8
3	Silver—citrate-capped (AgL1)	121.6
4	Silver—citrate- and tannic acid-capped (AgL2)	119.2
5	Selenium—chitosan-capped (Se1)	683.1
6	Selenium—BSA-capped (Se2)	642.8

**Table 2:** Elemental analysis of AgNP and SeNP suspensions were determined using ICP-OES. The table shows the measured concentrations of Ag and Se in different samples. <LOR indicates values below the limit of reporting.

Analyte	Ag1	Ag2	AgL1	AgL2	Se1	Se2	Limit of Reporting (mg/L)
	Conc. (mg/L)	Conc. (mg/L)	Conc. (mg/L)	Conc. (mg/L)	Conc. (mg/L)	Conc. (mg/L)	
Ag	57.44	265.8	121.6	119.2	0.678	0.113	0.050
Al	<LOR	0.0761	0.0728	0.0718	0.0707	0.069	0.050
B	<LOR	<LOR	<LOR	<LOR	159	162	0.050
Ba	<LOR	<LOR	<LOR	<LOR	<LOR	<LOR	0.010
Be	<LOR	<LOR	<LOR	<LOR	<LOR	<LOR	0.010
Ca	<LOR	0.0796	1.569	1.321	2.706	0.1536	0.050
Cd	<LOR	<LOR	<LOR	<LOR	<LOR	<LOR	0.050
Co	<LOR	<LOR	<LOR	<LOR	<LOR	<LOR	0.050
Cr	<LOR	<LOR	<LOR	<LOR	0.0519	<LOR	0.050
Cu	<LOR	<LOR	<LOR	<LOR	<LOR	<LOR	0.050
Fe	<LOR	<LOR	<LOR	<LOR	0.2791	<LOR	0.050
K	3.913	<LOR	<LOR	<LOR	<LOR	<LOR	0.250
Li	<LOR	<LOR	<LOR	<LOR	<LOR	<LOR	0.050
Mg	<LOR	<LOR	<LOR	<LOR	2.774	0.9465	0.250
Mn	<LOR	<LOR	<LOR	<LOR	<LOR	<LOR	0.050
Mo	<LOR	<LOR	<LOR	<LOR	<LOR	<LOR	0.250
Na	<LOR	111	66.07	81.34	658.8	675.9	0.250
Ni	<LOR	<LOR	<LOR	<LOR	<LOR	<LOR	0.050
P	<LOR	<LOR	<LOR	<LOR	<LOR	<LOR	0.500
Pb	<LOR	<LOR	<LOR	<LOR	<LOR	<LOR	0.250
S	<LOR	16.9	<LOR	<LOR	0.5252	42.16	0.500
Se	<LOR	<LOR	<LOR	<LOR	683.1	642.8	0.250
Si	<LOR	1.689	<LOR	<LOR	0.3339	<LOR	0.250
Sr	<LOR	<LOR	<LOR	<LOR	0.0169	<LOR	0.010
Ti	<LOR	<LOR	<LOR	<LOR	<LOR	<LOR	0.050
V	<LOR	<LOR	<LOR	<LOR	<LOR	<LOR	0.050
Zn	<LOR	0.9173	<LOR	<LOR	<LOR	<LOR	0.050
Zr	<LOR	<LOR	AA_01	<LOR	<LOR	<LOR	0.050

## Data Availability

Data is contained within the article.

## Abbreviations

ECC	Early Childhood Caries
SF	Silver Fluoride
NPs	Nanoparticles
AgNPs	Silver Nanoparticles
SeNPs	Selenium Nanoparticles
BSA	Bovine Serum Albumin
DI	Deionised (water)
AgNO_3_	Silver Nitrate
NaOH	Sodium Hydroxide
SPR	Surface Plasmon Resonance
Na_2_SeO_3_	Sodium Selenite
NaBH_4_	Sodium Borohydride
UV-Vis	Ultraviolet–Visible (Spectroscopy)
TEM	Transmission Electron Microscopy / Microscope
CARF	Central Analytical Research Facility
QUT	Queensland University of Technology
ICP-OES	Inductively Coupled Plasma Optical Emission Spectroscopy
ICP-MS	Inductively Coupled Plasma Mass Spectrometry
CRMs	Certified Reference Materials
Pb	Lead
Cd	Cadmium
Ag	Silver
Se	Selenium
